# Corneal Confocal Microscopy Findings in Neuro Lyme Disease: A Case Report

**DOI:** 10.3390/diagnostics12020343

**Published:** 2022-01-29

**Authors:** Pilar Cañadas, Montserrat García-Gonzalez, Rafael Cañones-Zafra, Miguel A. Teus

**Affiliations:** 1 Department of Optometry and Vision, School of Optics and Optometry, Complutense University of Madrid, 28037 Madrid, Spain; 2 Clínica Novovisión, P.° de la Castellana, 54, 28046 Madrid, Spain; montseggonz@gmail.com (M.G.-G.); rafael.canones@gmail.com (R.C.-Z.); miguelteus@gmail.com (M.A.T.); 3 Clínica Rementería, Calle Almagro, 36, 28010 Madrid, Spain; 4 Hospital Universitario “Príncipe de Asturias”, University of Alcalá, 28801 Alcala de Henares, Spain

**Keywords:** corneal confocal microscopy, Lyme disease, small fiber neuropathy, sub-basal nerve plexus

## Abstract

Neuro Lyme disease is caused by several bacteriae of the Borreliaceae family, such as Borrelia Miyamotoi. In late stages of illness, patients with Lyme disease may develop chronic neurologic symptoms such as cognitive disturbances or small fiber peripheral neuropathy. Confocal microscopy is a non-invasive method designed to evaluate the human cornea in vivo. Thus, all the corneal layers, including the cells and the sub-basal nerve plexus, can be easily visualized and analyzed. This is the first report of the morphology of small-fiber peripheral neuropathy analyzed by confocal microscopy in a patient diagnosed of neuro Lyme disease. The decrease in the number of unmyelinated sub-basal nerve fibers with abundant presence of dendritic cells (DC) in comparison with healthy corneas strongly supports the diagnosis of small fiber peripheral neuropathy in a case of neuroborreliosis disease.

## 1. Introduction

Lyme disease is caused by several spirochete bacteriae of the Borreliaceae family, such as *Borrelia (B.) Burgdorferi, B. Afzelii, B*. *Garinii* and *B. Miyamotoi*, among others [1,2]. Lyme disease is a zoonotic, vector-borne disease transmitted primarily by ticks. One of the new emerging Ixodes-borne Lyme diseases in Europe, as well as in the northern temperate climate zones of the world, is caused by *B. Miyamotoi* [3,4,5]. The most common sign of Lyme disease is an expanding red rash, known as erythema migrans, that appears at the site of the tick bite about one or two weeks after the bite occurred, although sometimes, the infection may occur without this cutaneous sign. A diagnosis of *B. Miyamotoi* disease should be considered in patients who experience fever attacks. Although *B. Miyamotoi* infection does not present specific symptoms of the relapsing fever group [6], the most reported clinical presentation of infection is a febrile illness consisting of fatigue, headache, chills, myalgias, arthralgias, and nausea [7,8].

In Europe, neurological conditions such as facial nerve palsy, meningitis, carditis [3], and peripheral neuropathy are some extracutaneous affections, even more so if the disease is not treated in its early stages [9]. 

Peripheral neuropathy and autonomic involvement are characterized by a selective alteration of small semi-demyelinated nerve fibers, as Aδ, and demyelinated C fibers. Most of the disautonomy symptoms include dry eye, dry mouth, orthostatic dizziness, cardiac palpitations, intestine alteration, etc. This condition could also be associated with other diseases such as fibromyalgia and diabetic neuropathy. Those peripheral neuropathies, in which small nerve fibers are affected, have been traditionally diagnosed by skin biopsies [10,11]. These biopsies show small fiber degeneration [11], but this is an invasive procedure that requires tissue processing and is time consuming.

The cornea, as one of the most innervated tissues in human body [12], receives heterogeneous sensory nerves from the ophthalmic branch of the trigeminal nerve. Functionally, sensory nerves vary in their chemical composition, electrophysiological properties, and response to excitation stimuli.

In addition to these sensory fibers, the cornea also receives a sparse supply of autonomic sympathetic nervous fibers, which originate in the cell bodies of the upper cervical ganglion [13,14,15], and autonomous parasympathetic nervous fibers (from the ciliary ganglion). The transparency of the stroma makes the cornea a good location to observe peripheral nerve fibers. It is also known that the cornea is composed of two types of nerve fiber: Aδ and C nerve fibers. Aδ are myelinated fibers of fast conduction, and they represent 20% of the total nerve fibers in the corneal sub-basal nerve plexus. The C fibers are unmyelinated and represent 80% of the total nerve corneal fibers [16].

In vivo corneal confocal microscopy (IVCM) is a non-invasive imaging technique that allows direct visualization of the corneal structure including the corneal sub-basal nerve plexus in vivo. IVCM can provide both a wide depth of focus and high resolution that permit the corneal evaluation at cellular level [17,18]; for this reason, IVCM is a very useful tool to identify small nerve fiber damage in several peripheral neuropathies [17,19,20]. To our knowledge, this is the first report of corneal nerve damage in a case of neuroborreliosis evaluated by IVCM.

## 2. Case Presentation

This is an observational, prospective study. It was performed in accordance with the tenets of the Helsinki Declaration of 1964. The patient gave informed consent for data collection and further publication of the study outcomes. Approval from the ethics committee was not required given both the observational nature of the study and the fact that usual clinical practice was followed. A 40 year-old woman, in 2004, presented with an episode of general malaise, joint pain, and fever for at least one week, which coincided with a trip to Greece in a rural environment. After this first episode, further episodes happened periodically with a duration of 5–7 days and with similar symptoms: arthralgia, tiredness, intense discomfort, dysthermic sensation, etc. The patient related these clinical episodes with different triggers such as the cold and minor physical effort. Since 2006, she referred to an intense and limiting intolerance to cold that manifested as hyperpathy, allodynia, and pain elicited by a minimal thermal stimulation.

In 2017, she also described vertigo symptoms that improved progressively and persistent neck pain after minimal physical exercise (after walking only a few meters).

In 2020, she was re-evaluated by internal medicine, and after a comprehensive exam, she was diagnosed with chronic fatigue secondary to a possible infection by *B. Miyamotoi*. The tests performed at that visit are shown in Table 1.

The hormonal study revealed very low levels of norepinephrine in prone position with a huge increase in response to standing, and normal levels of vasopressin in prone position with no change in response to standing.

Quantitative sensory test (QST) is a noninvasive way of quantifying sensory nerve function that is performed on one finger of the hand. The perception thresholds of touch or pressure, thermal (cold and warm), and pain (cold pain and heat pain) can be measured in a subjective way. In the patient, the results in each type of fibers were normal.

Sudomotor function (Electroconductance): The sudomotor function and the grade of asymmetry were evaluated in superior and inferior limbs by electroconductance. Electrochemical conductance (ESC) is expressed in microsiemens (µS) and is the relationship between the electricity produced and an applicate constant (<4 V). ESC evaluates the sudomotor local function. This sudomotor function expresses possible injuries in sympathetic fibers that innerve sweat glands. These long and thin demyelinated fibers in the autonomic nervous system are the first ones that cause damage in peripheral neuropathies. The evaluation of the magnitude of the response in symmetric locations of the body is important when characterizing a peripheral neuropathy. Both QST and ESC were in the low limits of normality and showed lesions in the small fibers that innerve sweat glands.

### 2.1. Ocular Examination

This patient was referred to us by her neurologist in order to obtain a corneal confocal microscopy of the sub-basal nerve plexus. Her ocular exploration was normal; only a diminished tear meniscus was found (1.7 mm) in both eyes, with no corneal or conjunctival alterations. The corneal estesiometry was normal in both eyes, and the fundus exam was also within normal limits.

### 2.2. Confocal Microscopy Findings

Confocal microscopy images were obtained using the Heidelberg Retina Tomograph II in combination with the Rostock Cornea Module (RCM) (Heidelberg Engineering, Heidelberg, Germany). The corneal nerve parameters evaluated were: main nerves’ density, total nerve length, nerve branches density, and dendritic cell density. The main nerves’ density expressed as number of nerve per mm^2^ (nerves/mm^2^) and the nerve length expressed as mean nerve length in µm/mm^2^ were measured using the plugin Neuron J (http://www.imagescience.org/meijering/software/neuronj/) (accessed on 20 December 2021). From Image J software (https://imagej.nih.gov/ij/) (accessed on 20 December 2021), which allows semi-automated tracing of nerve fibers and provides quantification. Nerve branches were manually numbered using the multipoint tool of the Image J software, and then the nerve branches density was calculated as the number of nerve branches per mm^2^ (nerves/mm^2^). Nerve branches were identified as the bifurcation of each main nerve. The dendritic cells, which can be identified in the sub-basal nerve plexus by their distinctive characteristics, bright cell bodies with dendritic structures, were manually numbered using the multipoint tool of the Image J software, and then the dendritic cell density was calculated (cells/mm^2^). In the process of dendritic cell quantification, the contrast of the image was modified in order to make the hyperreflective bodies of these cells more visible. This methodology is validated and previously described [21]. All these measurements were compared with the results of a control group of 20 healthy eyes of 20 subjects (2 males and 18 females), mean age: 45.56 ± 6.4 years (range, 36 to 52), and laterality (8 were left and 12 were right eyes). The methodology used in the control eyes to perform the confocal microscopy was the same used in the study eyes. These control patients had no other ocular or systemic disease, were non-contact lens wearers, used neither ocular medications nor artificial lubrication, were asymptomatic, and their ocular surface evaluation (slit-lamp examination, tear stability, ocular surface integrity, and tear production) was within normal limits.

The confocal microscopy findings are shown in Table 2.

**Figure 1:**
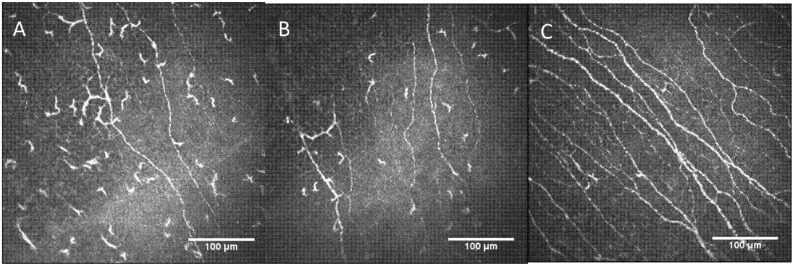
(**A**) Corneal nerve plexus in affected patient RE; (**B**) corneal nerve plexus in affected patient LE; (**C**) corneal nerve plexus image in a patient without systemic pathologies and no refractive surgery (control group). RE: right eye; LE: left eye.

The confocal images of corneal nerve plexus of both eyes of the affected patient versus a control group are shown in Figure 1.

**Figure 2:**
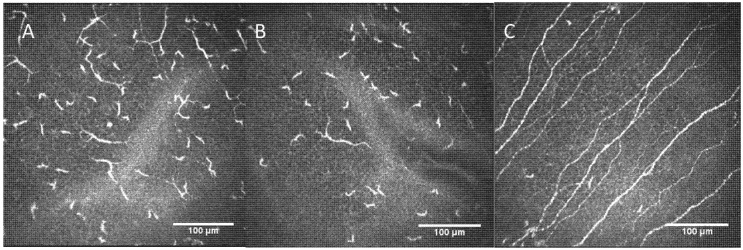
(**A**) Dendritic cell population in affected patient RE; (**B**) dendritic cell population in affected patient LE; (**C**) dendritic cell population in a patient without systemic pathologies or refractive surgery. RE: right eye; LE: left eye.

Dendritic cell density images in affected patient versus a control patient image are shown in Figure 2.

Regarding all the confocal results, the patient showed the criteria of involvement of small fiber neuropathy (SFN) in the context of an infectious episode (*B. Miyamotoi*). The presence of a decrease in unmyelinated fibers with abundant presence of dendritic cells (DCs) in the cornea supports this diagnosis. At hemodynamic level, very low blood pressure with low levels of norepinephrine in prone position also support the SFN. There was no sensory deficit on QST, but thermal allodynia persisted.

## 3. Discussion

One Lyme disease variant is caused by *B. Miyamotoi*. Regarding its epidemiology, there is limited knowledge of the full geographic distribution of human *B. Miyamotoi* infection, but it is likely to be similar to the Lyme disease variant caused by B. Burgdorferi [7]. Although the most commonly reported clinical presentation of *B. Miyamotoi* infection is a febrile illness with fatigue, headache, myalgia, arthralgia, etc. [22], in late stages, clinical presentation may show chronic neurologic symptoms, primarily cognitive disturbances or small fiber peripheral neuropathy (as spinal radicular pain or distal paresthesias or hypoesthesia) [23]. This type of Lyme disease is also called Lyme neuroborreliosis.

The cornea has two main types of nerve fibers: the A-delta (Aδ) and the C nerve fibers. The Aδ are high speed myelinated fibers (conduction average velocity of 6 m/s), and they represent about 20% of the total density of corneal afferent fibers. The C fibers are slower-conducting unmyelinated (conduction average velocity of less than 2 m/s) and are the most abundant fibers (around 80% of the total density of corneal afferent fibers) [24]. A-beta fibers are lacking in human corneas.

Regarding the type of stimuli that activate these nerve endings, there are three distinct classes of peripheral sensory nerve fibers: mechano-nociceptors (20% of all corneal sensory nerves; they are all Aδ fibers and they are activated by the mechanical contact with the cornea); polymodal nociceptors (the most abundant in the cornea, representing about 70% of all corneal sensory nerves; they are Aδ and C fibers and they are activated by mechanical, thermal and chemical—both exogenous molecules and endogenous inflammatory irritants—stimuli); and cold receptors (10% of all corneal sensory nerves; they are Aδ and C fibers and they are activated by tear film evaporation, cold air, and a decrease in the corneal temperature below 33 °C), and the application of cold solutions on the cornea [16,25,26,27].

Due to the high density of the corneal innervation, the cornea could be a target for SFN diagnosis. In fact, over the last decade, the quantification of small nerve fiber in the cornea using IVCM has been increasingly used for patients with small fiber dysautonomias [28,29], other peripheral neuropathies, such as diabetes [30], Charcot Marie Tooth [31], and multiple sclerosis [32]. All these conditions are characterized by a decrease in small-fiber density. This small nerve fiber loss may cause pain, dysesthesia, and/or autonomic dysfunction [33]. 

To our knowledge, this is the first report that shows the damage of the corneal nerves induced by the SFN secondary to neuroborreliosis using confocal microscopy. Both eyes of the patient showed a decrease in corneal main nerve density, nerve length, and nerve branch density, in comparison to healthy corneas.

In addition, another interesting finding of the current report was the higher number of DCs present in the cornea and their enlarged field area, in comparison to healthy eyes. The cornea, in particular, its basal epithelium, has an immunological attribute due the presence of DCs [34]. DCs are the main antigen-presenting immune cells in the cornea, which not only modulate innate and adaptive immune responses, but also contribute to the corneal nerves’ homeostasis maintenance [35]. In healthy eyes, DCs are characterized by having a cell body and dendrites, and they are located in the basal cell layer of the epithelium or in the sub-basal layer. DCs are considered to be in an immature state, with no obvious dendrites [36]. After trauma or an infection, the chemokines can lead to their activation and maturation. In our patient, affected with neuroborreliosis, the DCs revealed a high dendritic pattern. This finding has been described as a cellular maturation sign and is typically seen in autoimmune diseases or in chronic systemic inflammation [37]. This is the first report that shows the increase in corneal DCs density in neuroborreliosis.

## 4. Conclusions

In summary, the findings showed with IVCM supported the diagnosis of SFN in the context of neuroborreliosis by Borrellia Miyamotoi, with a comparable sensitivity and specificity to intra-epidermal nerve fiber density in skin biopsies [28,29,38], but with the advantage of being a non-invasive technique. In addition, IVCM could be an interesting tool for the follow-up of patients with neuroborreliosis or other SFN and for the evaluation of the efficacy of neurological treatments.

## Figures and Tables

**Table 1:** Blood tests, hormonal study, sensory test, and electro conductance performed.

Blood Tests	
Darkfield microscopy	Spirochetes forms-like compatible with Borrelia were detected.
Phage Borrelia qPCR	Borrelia Miyamotoi positive
Elisa e inmunoblot	Negatives
Elispot	Positive in B31/basal 20, Outer Surface Proteins (OSP)-mix/basal 13
**Basal Blood Pressure**	
Blood pressure (systolic/diastolic)	92/63 (mmHg)
Heart rate	71 beats per minute
**Biochemical Hormonal Specific Study**	
Hormone values in prone position (20 min)	
Noradrenaline: 62 pg/mL Antidiuretic hormone (vasopressin): <1.9 pg/mL	
Hormone values in standing position (3 min)	
Noradrenaline: 346 pg/mL Antidiuretic hormone (vasopressin): <1.9 pg/mL	
**Quantitative Sensory Test (QST)**	
Vibratory sensitivity (A-beta fibers)	Normal
Cold sensitivity: (A-delta fibers)	Normal
Hot sensitivity (C Fibers)	Normal
**Sudomotor Function. Electroconductance (ESC)**	
The mean ESC in inferior limbs	73 µS with a 0% of mean asymmetry
The mean ESC in superior limbs	72 µS with a 1% mean asymmetry

pg/mL: picograms/milliliter; mmHg: Mercury millimeters; µS: microsiemens.

**Table 2:** Confocal microscopy parameters in the affected patient compared to a control group.

Confocal Microscopy Parameters	Controls	Patient RE	Patient LE
Main nerves’ density (n/image ± SD)	10.5 ± 2.86	3	5
Total nerve length (mm/mm^2^ ± SD)	14.50 ± 3.6	6.64	8.27
Nerve branch density (n/image ± SD)	47.9 ± 26.2	0	2
Dendritic cells density (n/image ± SD)	31.3 ± 21.2	58	43

The n/image = number per image; RE: right eye; LE: left eye.
